# How Do Anchors' Characteristics Influence Consumers' Behavioural Intention in Livestream Shopping? A Moderated Chain-Mediation Explanatory Model

**DOI:** 10.3389/fpsyg.2021.730636

**Published:** 2021-09-28

**Authors:** Liangjie Zhu, Huiyao Li, Kun Nie, Chunmei Gu

**Affiliations:** School of Business Administration, Zhejiang Gongshang University, Hangzhou, China

**Keywords:** para-social interaction, anchors' characteristics, online interaction propensity, cognitive-affective system theory, livestream shopping

## Abstract

Livestream shopping has become the focus of current marketing practises, while theoretical research on it is still in initial stages. Thus, from the para-social interaction (PSI) theory perspective, this study draws on cognitive–affective system theory as an analytical framework to explore internal mechanisms of how anchors' characteristics influence consumer behavioural intentions in livestream shopping while considering the characteristics of consumer online interaction propensity. We conducted a survey questionnaire with a sample of 355 consumers who experienced livestream shopping and used structural equation modelling to assess their behavioural intentions. Our results reveal that anchors' physical attractiveness, social attractiveness, and professional ability influence consumers' intentions to follow the authors' suggestions and recommend anchors to others during live streams. PSI and affective trust in anchors are the chain-mediation mechanisms. Furthermore, consumers' online interaction propensity positively moderates the influence of anchors' characteristics on PSI and plays a moderating role on the whole chain mediation. However, this only affects anchors' physical attractiveness and social attractiveness while exert no effect on anchors' professional ability. This study advances the theoretical research on livestream shopping and provides practical inspiration for managers to develop more targeted livestream marketing strategies.

## Introduction

The proliferation of the Internet and the information and communication technologies have enabled the development of livestream shopping, which has gradually become an important means for businesses to improve their sales performance. In practise, a large number of “web celebrity” anchors have emerged on Chinese social e-commerce platforms. These anchors have a strong ability to “take goods” through a livestreaming platform to generate popularity and attract numerous “fans.” For example, Li Jiaqi and Via (two famous anchors in China) earned 9.2 billion yuan in livestream sales on the first day of Singles' Day in 2020.

Compared with more common practises, theoretical research on livestream shopping is still in its infancy. There are few studies on how anchor characteristics influence consumer behaviour. Peng et al. (2020) examined the relationship between anchors' physical characteristics and product sales and Park and Lin (2020) highlighted the fact that the matching degree between “web celebrity” anchors and livestreaming products can affect consumers' purchase intentions. However, these studies have two shortcomings. First, they do not systematically consider the influence of different characteristic dimensions of the anchors. Second, they regard anchors as “spokespersons” solely in livestream shopping, thereby ignoring anchors' influence in their interactive roles.

Unlike spokespersons in traditional advertising or TV shopping, livestreaming has reshaped the traditional communication model (Wang, 2021). Anchors interact with consumers in livestream shopping (Wongkitrungrueng and Assarut, 2020) and can display products to consumers more intuitively through tasting, trial play and trying out. Simultaneously, the livestreaming platform also enables consumers to interact with anchors in real-time through bullet screens, and anchors can provide personalised shopping guidance to consumers (Sun et al., 2019). The livestream shopping environment is characterised by a high level of interaction, which promotes the close interpersonal interaction between anchors and consumers (Al-Emadi and Yahia, 2020). Therefore, this study selects para-social interaction (PSI) theory for analysis, considering the characteristics of consumer online interaction propensity, and uses cognitive–affective system theory as an analytical framework to explore internal mechanisms of how anchors' characteristics influence consumers behavioural intentions in livestream shopping. PSI is an interactive reaction wherein audiences treat media characters as real individuals while using media (Horton and Wohl, 1956). Livestream shopping utilises real-time video interactive marketing, which creates a para-social environment. Online interaction propensity is the general propensity of an individual to interact with others in an online environment (Wiertz and De-Ruyter, 2007), which can be used to distinguish different individual characteristics in online communities.

## Literature Review and Hypotheses

### Livestream Shopping and Anchors

Livestream shopping is an online marketing method for sellers to show and sell products through a real-time streaming platform (Wongkitrungrueng and Assarut, 2020). Unlike traditional online shopping, consumers do not rely on pictures and texts but watch live streams and learn about products through a product display and the explanation of anchors in livestream shopping, where consumers can also ask questions and interact in real-time through bullet screens (Sun et al., 2019). This provides consumers with a more realistic product display environment, a more real-time online synchronous interaction and a social consumption scenario (Ang et al., 2018).

An anchor is a kind of Internet celebrity who relies on social media to become popular (Gerrath and Usrey, 2020; Chen et al., 2021). Compared to traditional celebrity endorsements by movie or athletic stars with a higher social status, many livestream shopping anchors are ordinary people, also known as “grassroots” celebrities (Wang, 2021). They usually have a high level of product knowledge and experience (López et al., 2021) and are more close to the life of an average consumer and therefore capable of becoming more interactive with followers and potential consumers (Al-Emadi and Yahia, 2020). During social media interactions, followers also may respond to the anchors, thus establishing a social relationship between them (Delbaere et al., 2020). In livestream shopping, anchor will give product display and shopping guidance, which not only establishes real-time interactive relationships with consumers but also generates a relatively higher number of buying conversations (Sun et al., 2019; Akdevelioglu and Kara, 2020).

### Para-Social Interaction: The Mediating Role of Anchors' Characteristics Influences on Consumer Behaviour

PSI was developed through the theory of social interaction, which is used to describe the relationship between the audience and media personalities in the media environment, such as fictional TV characters and news hosts (Horton and Wohl, 1956). PSI is also defined as a user's perceived interpersonal involvement with a media character through mediated communication (Chen et al., 2021). In PSI, the audience think that they have participated in a direct dialogue with a media character and may regard the media characters as “real friends” (Labrecque, 2014). In recent years, PSI has been widely applied in social media research and is used to explain the mediating variable of Internet users' online behaviour (Chen et al., 2021; Tsai et al., 2021).

In livestream shopping, anchors are the media characters, and the consumers who enter the livestreaming room are the audience. PSI will be formed between them during real-time livestream video interaction (Chen et al., 2021; Tsai et al., 2021). Some studies have shown that Internet celebrities' specialised knowledge, along with their personal appeal, promotes the formation of social relationships with consumers (Lin et al., 2018). In other words, anchors' characteristics in livestream shopping may influence consumer attitudes and behaviours through PSI. Furthermore, existing studies have divided anchor characteristics' dimensions into external “physical attractiveness,” internal “professional ability,” and interactive “social attractiveness” (Lee and Watkins, 2016). Specifically, physical attractiveness refers to consumers' perceptions on the degree of attractiveness of anchors' appearance. In relationship theory, appearance is an effective factor that determines whether people will be attracted (Finkel and Eastwick, 2015) and is also the prerequisite for enhancing influence in the minds of others (Lo, 2008). Professional ability refers to product knowledge, experience and other skills of anchors in the process of displaying products (Kim and Lennon, 2013). Consumers' perceptions of this inherent ability will increase consumers' trust in information sources (Peng et al., 2020). Social attractiveness refers to consumers' perceptions of the anchors' attractiveness in an interaction, which reflects a desire of the other party to become a friend or social partner in the interaction (McCroskey et al., 2006). People tend to have a more positive attitude towards socially attractive people (Ellegaard, 2012). In this study, consumers' behavioural intentions during livestreaming are divided into the intention to follow anchors' suggestions (the extent to which the audience listens, considers and implements the anchors' suggestions) and the intention to recommend anchors (audience's intentions to recommend anchors or livestreaming to others) (Casaló et al., 2020). Therefore, we hypothesise the following:

H1a: Anchors' physical attractiveness positively influences consumers' PSI and then positively affects consumers' intentions to follow anchors' suggestions and recommend anchors.
H1b: Anchors' professional ability positively influences consumers' PSI and then positively affects consumers' intentions to follow anchors' suggestions and recommend anchors.
H1c: Anchors' social attractiveness positively influences consumers' PSI and then positively affects consumers' intentions to follow anchors' suggestions and recommend anchors.

### Affective Trust: The Chain-Mediated Role

Mischel and Shoda (1995) proposed the cognitive–affective system theory, which states that when an individual is in a certain situation, the characteristics of the situation will activate some of its interconnected cognitive–affective units and then produce unique cognition, emotion and behaviour pertaining to the situation. Situational characteristics can be caused by external environments, such as social or interpersonal environments. Cognitive–affective unit is a series of psychological representation of individuals and is an internal psychological response to the self, others, goals or things that a person produces when facing the characteristics of the situation. The cognitive–affective unit can be a single cognitive or a single affective unit, and can also be a related cognitive and affective unit. The activation of the cognitive–affective unit influences consumer behaviour.

According to previous studies, PSI is a kind of relationship cognition which occurs when audience are building relationships with media characters (Ballantine and Martin, 2005; Hartmann and Glodhoorn, 2011). PSI can be understood as a cognitive unit. Affective trust is an affective connection with others or an affective dependence on others which be formed during the process of social or interpersonal communication (Schaubroeck et al., 2011). Affective trust evolves from trust, which includes two components, namely, cognitive trust at the rational level and affective trust at the perceptual level (Parayitam and Dooley, 2009). Cognitive trust is the judgement of the reliability of the opponent's ability and honesty (Johnson and Grayson, 2005), whereas affective trust is based on interpersonal communication and attraction (Chua et al., 2008). Accordingly, this study defines affective trust in anchors as an affective investment and audience's dependence on anchors. It belongs to the affective unit.

Building on the cognitive–affective system theory, the characteristics of anchors will affect the cognition of consumers' PSI, that is, the cognitive unit of consumers. When consumers' cognitive unit is activated, it will further affect whose affective unit. According to previous research, PSI embodies a kind of interpersonal relationship (Stern et al., 2007), and affective trust is formed during the process of interpersonal communication (Schaubroeck et al., 2011). Relationships can generate a kind of affective trust (Chua et al., 2008). Consumers' cognitive unit (the perception of PSI with anchors is activated) will further activate their affective unit (generate affective trust in anchors). PSI and affective trust in anchors constitute a chain-mediation mechanism. Specifically, anchors' characteristics will affect PSI, thereby activating consumers' affective trust in anchors, and then ultimately affect consumers' behavioural intentions regarding livestream shopping. Therefore, we hypothesise the following:

H2a: Anchors' physical attractiveness positively influences consumers' PSI, which further activates consumers' affective trust in anchors, and ultimately positively affects consumers' intentions to follow anchors' suggestions and recommend anchors.
H2b: Anchors' professional ability positively influences consumers' PSI, which further activates consumers' affective trust in anchors, and ultimately positively affects consumers' intentions to follow anchors' suggestions and recommend anchors.
H2c: Anchors' social attractiveness positively influences consumers' PSI, which further activates consumers' affective trust in anchors, and ultimately positively affects consumers' intentions to follow anchors' suggestions and recommend anchors.

### Online Interaction Propensity: The Moderating Effect

Online interaction propensity is the general propensity of an individual to interact with others in an online environment, it measures an individual's preference for participating in online interactions (Wiertz and De-Ruyter, 2007). Therefore, it can be used to distinguish different individual characteristics in an online community, for example, “active users” (individuals with a high propensity for online interaction) and “divers” (individuals with a low propensity for online interaction) among community members (Schlosser, 2005). Online interaction propensity is an inherent trait of individuals (Blazevic et al., 2014), which explains the differentiated online behaviour of people in the same online environment. Wiertz and De-Ruyter (2007) found that if consumers have a high propensity to interact online, they tend to make more intellectual contributions, for example, share and help. Similarly, Dessart (2017) indicated that online interaction propensity is positively related to social media engagement. Casaló et al. (2020) revealed that the influence of online opinion leaders on consumers' purchase intentions is also moderated by consumers' own online interaction propensity.

**Figure 1:**
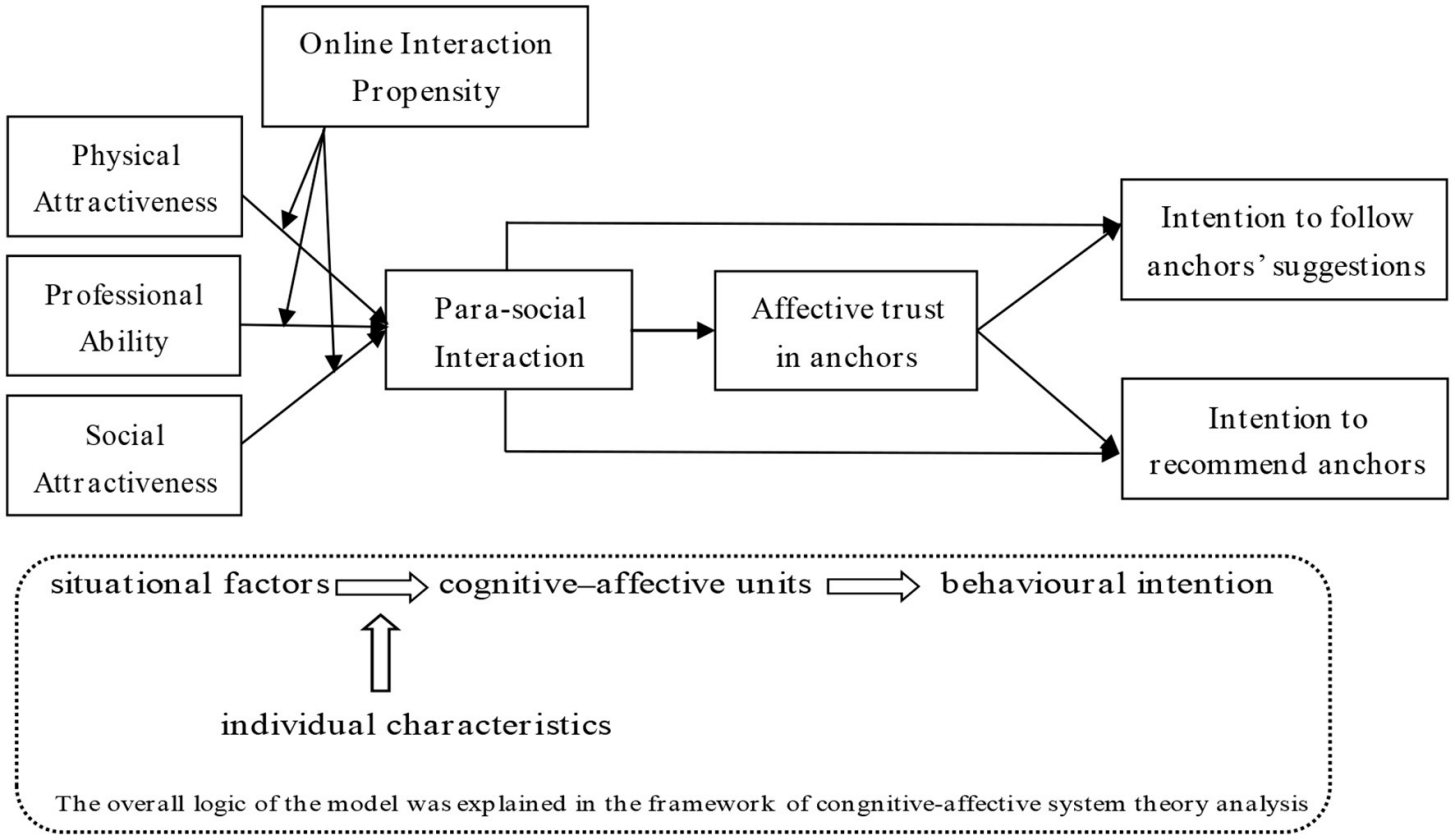
Theoretical model.

Studies in social psychology believed that there is an interaction between individual traits and situations (Kammrath et al., 2005). People engaging in social media interactions and relationships with media characters are influenced by their motivations and traits (Casaló et al., 2020; Qin, 2020). The conceptual connotation of online interaction propensity shows that consumers with high online interaction propensity are more willing to interact online and show more positive online relationships and behaviours (Wiertz and De-Ruyter, 2007). Therefore, the previous study offers further insights that when facing the same livestreaming situation, consumers' own online interaction propensity (individual traits) will affect PSI. Compared with consumers with low online interaction propensity, those with high online interaction propensity are more likely to form consumers' PSI. Online interaction propensity moderates the influence of anchors' characteristics on consumers' PSI. Thus, according to the cognitive–affective system theory, PSI and affective trust in anchors constitute a mutual influence relationship of cognitive–affective units. In other words, the impact of the interaction between online interaction propensity and the anchors' characteristic on consumers' PSI will further affect consumers' affective trust in anchors, thereby promoting consumers' intentions to act more positively. Therefore, we hypothesise the following:

H3a: Consumers' online interaction propensity positively moderates their PSI and affective trust in anchors in the chain mediation relation between anchors' physical attractiveness and consumers' intentions to follow anchors' suggestions and recommend anchors.
H3b: Consumers' online interaction propensity positively moderates their PSI and affective trust in anchors in the chain mediation relation between anchors' professional ability and consumers' intentions to follow anchors' suggestions and recommend anchors.
H3c: Consumers' online interaction propensity positively moderates their PSI and affective trust in anchors in the chain mediation relation between anchors' social attractiveness and consumers' intentions to follow anchors' suggestions and recommend anchors (Figure 1).

## Research Method

### Measurement Scale and Questionnaire Collection

The source of the scale required for this study is as follows. The items of anchors' physical and social attractiveness are adapted from Lee and Watkins (2016). The items of anchors' professional ability are adapted from Ohanian (1990). For mediation variables, the items of PSI are adapted from Lee and Watkins (2016). The items of affective trust in anchors are adapted from McAllister (1995). For the moderation variables, the items of online interaction propensity are adapted from Casaló et al. (2010). For outcome variables, the items of the intention to follow anchors' suggestions and recommend anchors are adapted from Casaló et al. (2020). The specific measurement items of each scale are shown in Table 1. To avoid the problem of homology variance, a 5-level Likert scale was used to measure PSI and affective trust in anchors and a 7-level Likert scale was used for the remaining variables.

**Table 1:** Variable measurement scale.

**Variable**	**Measuring project**	**Factor loading**
Physical attractiveness	I think this anchor is very attractive physically.	0.97
	I think this anchor is quite pretty/handsome.	0.95
	I think this anchor is very sexy.	0.76
Professional ability	I think this anchor has professional skills.	0.80
	I think this anchor has special skills.	0.72
	I think this anchor has rich experience in using the recommended products.	0.82
	I think this anchor has professional knowledge.	0.88
Social attractiveness	I think this anchor could be a friend of mine.	0.88
	I think it's very easy to communicate with this anchor.	0.73
	I would like to have a friendly chat with this anchor.	0.93
	I think we can form a personal friendship with each other.	0.86
Para-social interaction	I look forward to watching the livestreaming on his/her live channel.	0.80
	If this anchor appeared on another livestreaming platform, I would watch him/her living.	0.76
	When I'm watching this anchor, I feel as if I am part of his/her group.	0.77
	I think this anchor is like an old friend.	0.79
	I would like to meet this anchor in person.	0.71
	If there was information about this anchor online, I would read it.	0.71
	This anchor makes me feel comfortable, as if I am with a friend.	0.71
	When this anchor shows how his/her feels about a brand, it influences the viewers' perception about the brand.	0.64
Affective trust in anchors	I would like to share my thoughts or feelings with this anchor.	0.86
	I think this anchor is willing to listen to my problems or difficulties.	0.93
	I feel a sense of loss when I couldn't see the live streaming of this anchor.	0.91
	I think this anchor will care about and respond to my needs.	0.89
Intention to follow advice	I think it would be comfortable to use what this anchor recommends.	0.91
	I would not hesitate to take into account the suggestions about product purchases given by this anchor.	0.82
	I would feel secure in following the suggestions about product purchases made by this anchor.	0.92
	I would rely on the recommendations about product purchases made by this anchor.	0.80

*Significance level p < 0.001; χ^2^ = 1,047.23; χ^2^/df = 3.39, RMSEA = 0.08, SRMR = 0.07, CFI = 0.91; TLI = 0.90; The intention to recommend anchors adopts the scale of Casaló et al. (2020), which has only one item and belongs to an observation variable; therefore, reliability and validity tests are not conducted*.

Regarding data collection, the professional online platform “Sojump” was used to distribute and collect data nationwide. To make the data real and effective, the following two steps were adopted to strictly identify and delete invalid questionnaires. The first step is to delete invalid questionnaires with a “no” answer to the question, “Have you ever watched a livestreaming?” The second step is to delete any questionnaire that took little time to complete. After the unqualified questionnaires were eliminated through the above steps, a total of 355 valid questionnaires were collected. Men accounted for 35.2% and women 64.8%. Of the samples, 41.7%, 27.6%, 22.2%, and 8.5% were aged 18–24, 25–30, 31–36, and ≥37 years, respectively. The people who watched the livestreaming on average for >2, 1–2 and <1 times per month accounted for 38.8%, 37.5%, and 23.7%, respectively.

### Reliability and Validity

Before verifying the model, reliability and validity tests were conducted on the relevant scales. This study uses Cronbach's alpha and composite reliability (CR) to test the model's reliability. Table 2 shows that Cronbach's alpha values exceed the required value of 0.7, which satisfies the reliability requirement. Results show that CR of all the scales is above 0.8, and the reliability of the scales is good. This study further conducted model confirmatory factor analysis. The results show that the model fitting indicators are χ^2^ = 1047.23; χ^2^/df = 3.39, RMSEA = 0.08, SRMR = 0.07, CFI = 0.91; TLI = 0.90. The model has reached an acceptable level and has good construct validity. Additionally, we use the average variance extracted (AVE) and item loading to test for convergent validity. The AVE values of all constructs are above 0.5, which exceeds the accepted level, and all the item loadings exceed the required value of 0.7. The results thus show that convergent validity is also satisfied, and the model variables have good convergence validity. The correlation coefficient between each variable is less than the square root of the AVE, indicating that the model variables have good discriminative validity. The results are shown in Tables 1, 2.

**Table 2:** AVEs, CRs, and correlation coefficient between variables (*N* = 355).

**Constructs**	**Cronbach's Alpha**	**AVE**	**CR**	**MEAN**	**SD**	**PHA**	**PA**	**SA**	**PSI**	**ATA**	**IFA**
PHA	0.92	0.81	0.93	4.75	1.25	**0.90**					
PA	0.88	0.65	0.88	4.95	1.23	0.48^***^	**0.81**				
SA	0.91	0.73	0.92	3.63	1.46	0.37^***^	0.48^***^	**0.85**			
PSI	0.91	0.56	0.91	3.22	0.82	0.48^***^	0.63^***^	0.73^***^	**0.75**		
ATA	0.94	0.81	0.94	3.43	0.85	0.44^***^	0.70^***^	0.55^***^	0.74^***^	**0.90**	
IFA	0.92	0.75	0.92	4.52	1.23	0.31^***^	0.57^***^	0.45^***^	0.64^***^	0.77^***^	**0.87**

*Significance level*

*** *p < 0.005. PHA, physical attractiveness; PA, professional ability; SA, social attractiveness; PSI, para-social interaction; ATA, affective trust in anchors; IFA, intentions to follow anchors' suggestions. Diagonal elements represent the square root of AVE*.

This study adopts Harman's single factor method to test whether there is a common method deviation between the research variables. The results show that the overall variance explained for factors with a characteristic root above 1 is 72.843%, and the first principal component is 48.16%, which does not exceed the critical value of 50%. This shows that there is no homology error between the model variables.

### Mediating Effects

We used Mplus7.4 software to test the significance of the path coefficient of the constructed structural equation model and bootstrapping to test the mediating effect. The sample size was selected as 1,000. Among them, the independent variables are physical attractiveness (X1), professional ability (X2), and social attractiveness (X3). The mediating variables are PSI (M1) and affective trust in anchors (M2); the dependent variables are the intentions to follow anchors' suggestions (Y1) and recommend anchors (Y2). To improve the accuracy and reliability of the results, variables such as livestreaming frequency, age and gender were controlled. The analysis of the results obtained is as follows.

First, the direct path coefficients of anchors' physical attractiveness, professional ability and social attractiveness to consumers' intentions to follow anchors' suggestions and recommend anchors are not significant (*p* > 0.05). This shows that these features cannot directly affect consumers' intentions to follow anchors' suggestions and recommend anchors.

Second, anchors' physical attractiveness, professional ability and social attractiveness influence the intentions of consumers to follow anchors' suggestions and recommend anchors through PSI (M1), specifically, anchors' physical attractiveness (β = 0.135, *p* < 0.001), professional ability (β = 0.192, *p* < 0.001) and social attractiveness (β = 0.257, *p* < 0.001). Additionally, PSI impacts the intention to follow anchors' suggestions (β = 0.281, *p* < 0.05) and recommend anchors (β = 0.697, *p* < 0.001), which indicates that consumers' PSI has a positive effect on their intentions. Table 3 shows the results of the mediating effects assessment test. In other words, the mediating effect values of PSI between physical attractiveness, professional ability, social attractiveness, and intentions to follow anchors' suggestions and recommend anchors are 0.071 (*p* < 0.01) and 0.039 (*p* < 0.05), 0.101 (*p* < 0.01) and 0.056 (*p* < 0.05) and 0.161 (*p* < 0.001) and 0.089 (*p* < 0.05), respectively. The 95% confidence intervals of bootstrap (*n* = 1,000) are [0.031, 0.116] and [0.008, 0.077], [0.044, 0.174] and [0.013, 0.109] and [0.083, 0.250] and [0.023, 0.172], respectively none contains 0, indicating that the complete mediating effect is significant, and H1 is supported.

**Table 3:** Testing the mediating effect.

**Path**	**Indirect effect estimation (Standardisation)**		**95% confidence interval**	
			**Lower limit**	**Upper limit**
X1 → Y1	Total indirect effect	0.110^***^	0.049	0.170
	**Specific indirect effect decomposition**			
	X1 → M1 → Y1	0.071^**^	0.031	0.116
	X1 → M1 → M2 → Y1	0.031^**^	0.011	0.056
X2 → Y1	Total indirect effect	0.248^***^	0.159	0.337
	**Specific indirect effect decomposition**			
	X2 → M1 → Y1	0.101^**^	0.044	0.174
	X2 → M1 → M2 → Y1	0.044^**^	0.016	0.074
X3 → Y1	Total indirect effect	0.242^***^	0.163	0.329
	**Specific indirect effect decomposition**			
	X3 → M1 → Y1	0.161^***^	0.083	0.250
	X3 → M1 → M2 → Y1	0.070^**^	0.030	0.108
X1 → Y2	Total indirect effect	0.107^***^	0.034	0.179
	**Specific indirect effect decomposition**			
	X1 → M1 → Y2	0.039^*^	0.008	0.077
	X1 → M1 → M2 → Y2	0.054^**^	0.024	0.086
X2 → Y2	Total indirect effect	0.312^***^	0.241	0.386
	**Specific indirect effect decomposition**			
	X2 → M1 → Y2	0.056^*^	0.013	0.109
	X2 → M1 → M2 → Y2	0.076^***^	0.040	0.115
X3 → Y2	Total indirect effect	0.229^***^	0.136	0.335
	**Specific indirect effect decomposition**			
	X3 → M1 → Y2	0.089^*^	0.023	0.172
	X3 → M1 → M2 → Y2	0.121^***^	0.078	0.174

*Significance level*

* *p < 0.05*,

** *p < 0.01, and*

*** *p < 0.005. X1 is physical attractiveness, X2 is professional ability, X3 is social attractiveness, M1 is PSI, M2 is affective trust in anchors, Y1 is intentions to follow anchors' suggestions and Y2 is intentions to recommend anchors*.

Third, PSI positively impacts consumers' affective trust in anchors (β = 0.477, *p* < 0.001), and affective trust in anchors positively impacts intentions to follow anchors' suggestions (β = 0.791, *p* < 0.001) and recommend anchors (β = 0.624, *p* < 0.001).

Fourth, PSI and affective trust in anchors mediate the influence of physical attractiveness, professional ability and social attractiveness on intentions to follow anchors' suggestions and recommend anchors. Specifically, the mediating effect values of PSI and affective trust in anchors in physical attractiveness, professional ability, social attractiveness and intentions to follow anchors' suggestions and recommend anchors are 0.031 (*p* < 0.01) and 0.054 (*p* < 0.01), 0.044 (*p* < 0.01) and 0.076 (*p* < 0.001) and 0.070 (*p* < 0.01) and 0.121 (*p* < 0.001), respectively. The 95% confidence intervals of bootstrap (*n* = 1,000) are [0.011, 0.056] and [0.024, 0.080], [0.016, 0.074] and [0.040, 0.115] and [0.030, 0.108] and [0.078, 0.174], respectively none contains 0, indicating that the chain-mediating effect is significant; thus, H2 is supported. The results are shown in Table 3.

### Moderating Effects

To test the moderating effect of online interaction propensity, the moderating chain-mediation model algorithm proposed by Stride et al. (2015) was used. The results are as follows.

First, the interactive items between physical attractiveness and online interaction propensity (β = 0.091, *p* < 0.05) and that between social attractiveness and online interaction propensity (β = 0.105, *p* < 0.05) are positively associated with PSI. However, the interactive items of professional ability and online interaction propensity are not positively associated with PSI (β = 0.014, *p* > 0.05). The result indicates that online interaction propensity significantly moderates the impact of physical attractiveness and social attractiveness, rather than professional ability, on PSI.

Second, the moderated chain-mediation model with the interactive items is moderated in the first stage. Therefore, this study uses the coefficient product method to analyse the moderated chain-mediating effect, that is, testing the significance of the path coefficient product between the interactive items and mediation variables to determine whether the mediating effect is significant. In particular, a1 is used to denote the path coefficient between physical attractiveness (X1) and PSI, a2 is used to denote the path coefficient between online interaction propensity and PSI (M1). a3 is used to denote social attraction (X3), which is the path coefficient between online interaction propensity and PSI (M1); d1 represents the path coefficient between PSI (M1) and affective trust in anchors (M2). b2 and b4 are used to represent the path coefficients between affective trust in anchors (M2) and their intentions to follow anchors' suggestions (Y1) and recommend anchors (Y2). Statistical results show the following: a1 × d1 × b2 = 0.044 (*p* < 0.05), a3 × d1 × b2 = 0.062 (*p* < 0.01), a1 × d1 × b4 = 0.055 (*p* < 0.001), and a3 × d1 × b4 = 0.079 (*p* < 0.001). In other words, online interaction propensity moderates the chain-mediating effect.

At the same time, this study further validates the difference analysis method proposed by Edwards and Lambert (2007) by directly testing the significance of the mediating effect and thus determines whether the mediating effect is significant. This study simultaneously tests the online interaction propensity associated with PSI and affective trust in anchors, which plays a moderating role in the chain-mediating effect between physical attractiveness, social attractiveness and intentions to follow anchors' suggestions and recommend anchors. Specifically, the moderating effect of different online interaction propensity (one standard deviation below and above the mean) levels is analysed by the bootstrap method.

When the online interaction propensity is low, the mediating effect values of physical attractiveness and social attractiveness through PSI are 0.035 (*p* < 0.05) and 0.028 (*p* < 0.05), respectively. Meanwhile, the mediating effect values of affective trust in anchors that impacts the intentions to follow anchors' suggestion and recommend anchors are 0.096 (*p* < 0.001) and 0.075 (*p* < 0.01), respectively. Moreover, the 95% confidence intervals of bootstrap (*n* = 1000) are [0.004, 0.069] and [0.004, 0.062], [0.057, 0.148] and [0.033, 0.132], respectively.

When the online interaction propensity is high, the mediating effect values of physical attractiveness and social attractiveness through PSI are 0.075 (*p* < 0.001) and 0.059 (*p* < 0.01), respectively. Meanwhile, the mediating effects of affective trust in anchors that impacts intentions to follow anchors' suggestion and recommend anchors are 0.063 (*p* < 0.01) and 0.049 (*p* < 0.01), respectively. Moreover, the 95% confidence intervals of bootstrap (*n* = 1000) are [0.041, 0.125] and [0.025, 0.119] and [0.034, 0.107] and [0.022, 0.093], respectively. None of the above confidence intervals contain 0, indicating that the moderated chain-mediating effects corresponding to the respective variables are all significant.

The aforementioned data show a significant difference between the indirect effects of the chain-mediation path when the online interaction propensity is low vs. high. This suggests that when consumers' online interaction propensity is high, the association between the chain-mediating effect on anchors' physical attractiveness and social attractiveness through PSI and affective trust in anchors impacting consumers' intentions to follow anchors' suggestions and recommend anchors is significantly enhanced. In other words, consumers' online interaction propensity positively moderates the chain-mediating effect between PSI and the affective trust in anchors between anchors' characteristics (physical attractiveness and social attractiveness) and consumers' intentions to behave positively. However, it does not work in the dimension of anchors' professional ability characteristics. Thus, H3 is partially supported. The results are presented in Table 4.

**Table 4:** Moderated chain-mediated effect analysis.

**Moderating variable**	**Path**	**Indirect effect**	**95% confidence interval**	
			**Lower limit**	**Upper limit**
Low online interaction propensity	X1 → M1 → M2 → Y2	0.035^*^	0.004	0.069
	X3 → M1 → M2 → Y2	0.096^***^	0.057	0.148
	X1 → M1 → M2 → Y1	0.028^*^	0.004	0.062
	X3 → M1 → M2 → Y1	0.075^**^	0.033	0.132
High online interaction propensity	X1 → M1 → M2 → Y2	0.075^***^	0.041	0.125
	X3 → M1 → M2 → Y2	0.063^**^	0.043	0.107
	X1 → M1 → M2 → Y1	0.059^**^	0.025	0.119
	X3 → M1 → M2 → Y1	0.049^**^	0.022	0.093
Discrepancy	X1 → M1 → M2 → Y2	0.040^***^	0.037	0.059
	X3 → M1 → M2 → Y2	−0.033^***^	−0.014	−0.041
	X1 → M1 → M2 → Y1	0.031^***^	0.021	0.057
	X3 → M1 → M2 → Y1	−0.026^***^	−0.011	−0.039

*Significance level*

* *p < 0.05*,

** *p < 0.01, and*

*** *p < 0.005. X1 is physical attractiveness, X2 is professional ability, X3 is social attractiveness, M1 is PSI, M2 is affective trust in anchors, Y1 is intentions to follow anchors' suggestions, and Y2 is intentions to recommend anchors*.

## Research Conclusion and Discussion

### Research Conclusions

This study empirically proposed and verified a research model to explore the internal mechanism of how anchors' characteristics influence consumer behavioural intentions in livestream shopping.

First, our results confirmed that anchors' physical attractiveness, professional ability and social attractiveness indirectly influence consumers' intentions to follow anchors' suggestions and recommend them to others. The mediation mechanism is that the characteristics of anchors in these three aspects positively affect consumers' PSI. These characteristics further influence consumers' affective trust in anchors positively, thereby promoting consumers' intentions to behave positively. PSI and affective trust in anchors are the chain-mediation mechanisms by which anchors' characteristics influence consumers' intentions to follow anchors' suggestions and recommend them.

Second, this study found that the individual characteristics of consumers' online interaction propensity moderate this effect. Specifically, it positively moderates the chain-mediating effect of consumers' PSI and affective trust in anchors between anchors' characteristics (physical attractiveness and social attractiveness) and consumers' positive behavioural intentions. However, this moderating effect only works on the two characteristic dimensions, namely, anchors' physical attractiveness and social attractiveness, it does not work on anchors' professional ability. The reason why moderating effect only works on anchors' physical attractiveness and social attractiveness is because online interaction propensity essentially reflecting an individual's extraverted or introverted characteristics, which can, to a greater extent, explain individuals' cognition, emotion and behaviour in situations, or features that closely related to interpersonal relationships. From the perspective of conceptual connotation, physical attractiveness and social attractiveness are derived from social relationship theory (Finkel and Eastwick, 2015), which describes the characteristics in the context of interpersonal communication. Professional ability is rooted in cognitive psychology (Johnson and Grayson, 2005; Kim and Lennon, 2013), which is a kind of human cognition that does not reflect the conceptual connotation of interpersonal relationship orientation. Therefore, the online interaction propensity does not moderate the impact of professional ability on PSI.

### Theoretical Contributions

First, this study explains the influencing mechanism of livestream shopping on consumer behaviour from a new theoretical perspective. Although a small number of existing studies have explored how the characteristics of anchors influence consumer behaviour, the explanation mechanism of these studies has been relatively simple. For example, the match between anchor characteristics and live content is used to explain the mechanism of livestream shopping (Park and Lin, 2020), which is also essentially applicable to research on the matching relationship between spokespersons and endorsement products in a generalised context. Besides, the livestream shopping environment is often regarded as a background rather than a special situation or environmental characteristics. This may lead to the general applicability of its research conclusions and lack of pertinence to the characteristics of the live-marketing environment. Given the shortcomings of existing research, the interpretation mechanism proposed in this study more explicitly combines the characteristics of the livestream shopping environment and improves the pertinence of research conclusions. This also more comprehensively reveals the “black box” of consumer behaviour in the livestream shopping process and enriches the theoretical research results in livestream marketing.

Second, this research advances the study of PSI theory. Although scholars have recently begun to use PSI theory to explain consumer behaviour in social media environments (Ballantine and Martin, 2005; Labrecque, 2014; Gong and Li, 2017), numerous studies related to PSI theory still focus on traditional media situations (Auter and Palmgreen, 2000; Yuksel and Labrecque, 2016). This study enriches the research of PSI theory in the context of new media. More importantly, although some studies have explored the antecedent effects of media characteristics on the formation of PSI (Schramm and Hartmann, 2008; Knoll et al., 2015), these studies have hardly considered the boundary conditions for the formation of PSI. Moreover, this study introduces online interaction propensity as a characteristic variable of consumers to further explain the formation mechanism. The results of this study suggest that the formation of PSI by media personas is not always effective, which further advances the research on the mechanism of the formation of PSI.

### Practical Implications

First, this study provides strategic inspiration for companies to select and train anchors. The study findings reveal that anchors' physical attractiveness, professional ability and social attractiveness can influence consumers' intention to follow suggestions and recommend anchors to others in livestream shopping. This suggests that companies should prefer those with an excellent external image, high product expertise and rich interactive skills when selecting anchors. For anchor training institutions, special attention should be paid to shape the three aforementioned characteristics of anchors. Furthermore, this research finds that although anchors' physical attractiveness, professional ability and social attractiveness all impact consumers' intentions to behave positively, physical attractiveness and social attractiveness are affected by consumers' online interaction propensity. This provides insights for companies that anchors' professional ability is the most basic and often the most important characteristic dimension during livestreaming. Therefore, full consideration, such as detailed and enough professional ability training, also should be paid no matter when anchors are newly employed or worked for the company for a period of time.

Second, this study provides practical inspiration for developing differentiated livestream shopping strategies. It shows that consumers' personal traits of online interaction tend to moderate the effect of anchors in the livestream shopping scenario. Specifically, anchor characteristics have a more effective influence on the behavioural intentions of consumers with high (vs. low) online interaction propensity. This inspires anchors to discover and guide consumers with high online interaction propensity well during livestreaming. This is the highest quality available resource in the livestreaming room. In terms of marketing strategy, this type of consumer can be developed into a key opinion leader in the livestreaming room by emphasising interactive benefits (e.g., reputation and title). For consumers with low online interaction propensity, it may be more effective for anchors to emphasise economic benefits (e.g., discounts) than interactive benefits.

Third, the conclusion of this study also reflects the current live-marketing practise wherein many consumers participating in livestream shopping are young “Internet natives.” Such consumers have a higher tendency to interact online, therefore, the effects of livestream shopping tend to be more prominent. This inspires companies to highlight the role of anchors when their target customers are “Internet natives” in choosing livestreaming strategies. Regarding the choice of anchors, companies can focus on the “head” or “waist” anchors. In other words, when consumers with a relatively low online interaction propensity joined the livestreaming, the impact of anchors may be relatively low. At this time, companies should start from the maximisation of marketing efficiency and choose some “experienced” anchors.

### Limitation and Future Research

Despite some novel findings provided by this study, it still has some shortcomings. First, in this study, we divided anchor characteristics into physical attractiveness, professional ability and social attractiveness. But these three characteristics are derived from the description of media characters in the traditional media environment. Second, this study introduces online interaction propensity as a boundary condition for interpretation. Although this fits well with the online livestreaming situation, the type of the livestreaming product is also an important factor that moderates the effect of livestream shopping which also was not considered in our study.

There are two directions worth expanding in future research. First, the influence of anchors' characteristics on consumers' livestream shopping behaviours can be discussed from other perspectives, such as the body language and language style of anchors. Second, in terms of research methods, real longitudinal data or sales data could be used to explore the relationship between different characteristics of anchors and consumer behaviours in livestream shopping.

## Data Availability Statement

The original contributions presented in the study are included in the article/supplementary material, further inquiries can be directed to the corresponding author/s.

## Author Contributions

LZ was in charge of the paper writing. HL was in charge of data collection and processing. KN and CG gave suggestions for the revision of the paper. All authors contributed to the article and approved the submitted version.

## Funding

This research was supported by the Zhejiang Natural Science Foundation (LQ21G020002) and National Social Science Foundation of China (20BGL058).

## Conflict of Interest

The authors declare that the research was conducted in the absence of any commercial or financial relationships that could be construed as a potential conflict of interest.

## Publisher's Note

All claims expressed in this article are solely those of the authors and do not necessarily represent those of their affiliated organizations, or those of the publisher, the editors and the reviewers. Any product that may be evaluated in this article, or claim that may be made by its manufacturer, is not guaranteed or endorsed by the publisher.
